# Wine cup stoma anastomosis after extended sleeve lobectomy for central-type squamous cell lung cancer

**DOI:** 10.1186/s13019-019-0857-3

**Published:** 2019-02-12

**Authors:** Mitsunori Higuchi, Masayuki Watanabe, Kotaro Endo, Ikuro Oshibe, Nobutoshi Soeta, Takuro Saito, Hiroshi Hojo, Hiroyuki Suzuki

**Affiliations:** 1 Department of Thoracic Surgery, Aizu Medical Center, Fukushima Medical University, Aizuwakamatsu, 969-3492 Japan; 2 Department of Chest Surgery, Fukushima Medical University School of Medicine, Fukushima, 960-1295 Japan; 3 Department of Surgery, Aizu Medical Center, Fukushima Medical University, Aizuwakamatsu, 969-3492 Japan; 4 Department of Pathology, Aizu Medical Center, Fukushima Medical University, Aizuwakamatsu, 969-3492 Japan

**Keywords:** Extended sleeve lobectomy, Wine cup stoma, Bronchial anastomosis, Central-type lung cancer

## Abstract

**Background:**

Extended sleeve lobectomy is rarely applied to pulmonary surgery for primary lung cancer to avoid a pneumonectomy. As there is a size discrepancy between main bronchus and peripheral bronchus, ingenuity to improve anastomosis is required in the bronchoplasty. We report herein a case in which successful reconstruction of extended sleeve lobectomy with bronchial wall flap.

**Case presentation:**

We report on a 64-year-old man suffering from hemoptysis, cough, mild fever and dyspnea. His computed tomography (CT) scan showed solid tumor of 40 mm in diameter in left lower bronchus, which obstructed the lower bronchus and caused obstructive pneumonia of left lower lobe and expanded to second carina and pulmonary artery. His bronchoscopy showed that tumor was exposed in the bronchial lumen and infiltrated to left main bronchus and upper bronchus even though the scope could pass through the exposed tumor of upper bronchus. Transbronchial lung biopsy showed squamous cell carcinoma. He had undergone left sleeve lingular segmentectomy and left lower lobectomy. Reconstruction was performed with bronchial wall flap. Pathological findings revealed pT3N0M0 stage IIB according to UICC 8th edition. Postoperative bronchoscopic findings showed no troubles at the anastomotic site. He has been well for eighteen months without recurrence after surgery.

**Conclusions:**

We experienced a successful case who was reconstructed with bronchial wall flap (wine cup stoma) after extended sleeve lobectomy. This technique might be also useful for other types of extended sleeve lobectomy and lung transplantation to adjust caliber changes of bronchi.

## Background

Central-type lung cancer sometimes invades bronchial openings and/or the pulmonary artery (PA). For these patients, lobectomy/segmentectomy with bronchoplasty or PA angioplasty is often preferred. This surgery sometimes requires simultaneous reconstruction of the airways and/or blood vessels. On the other hand, pneumonectomy for lung cancer is reportedly associated with significant morbidity and mortality [1–3], including postpneumonectomy lung edema, adult respiratory distress syndrome, bronchopleural fistula, and postpneumonectomy syndrome [3]. Previous reports have already shown that lobectomy with bronchoplasty or angioplasty is a more feasible surgery than pneumonectomy for central-type non-small cell lung cancer (NSCLC). An extended sleeve lobectomy is rarely attempted to avoid pneumonectomy for patients with primary lung cancer. This atypical bronchoplasty requires some technical skills because there is a large size discrepancy between the two bronchial stumps. Herein we report successfully implementation of an extended sleeve lobectomy with bronchial wall flap technique, “wine cup anastomosis”.

## Case presentation

**Fig. 1:**
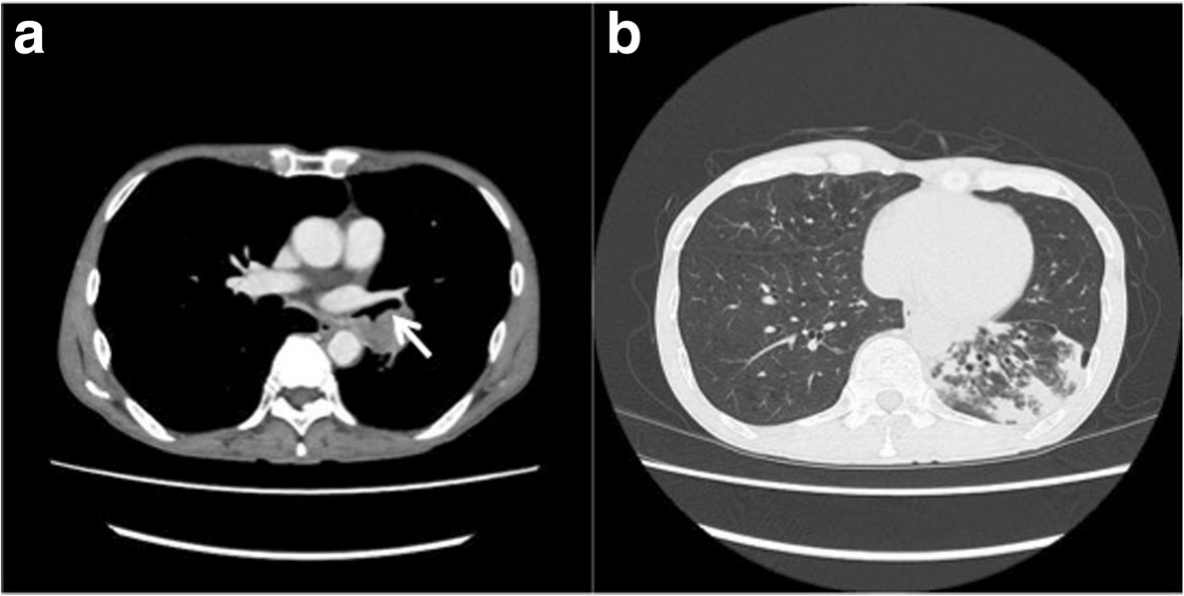
Computed tomography (CT) scan showed solid tumor of 40 mm in diameter in left lower bronchus which involved lingular division bronchus (solid arrow) (**a**), which also obstructed the lower bronchus and caused obstructive pneumonia of left lower lobe (**b**)

**Fig. 2:**
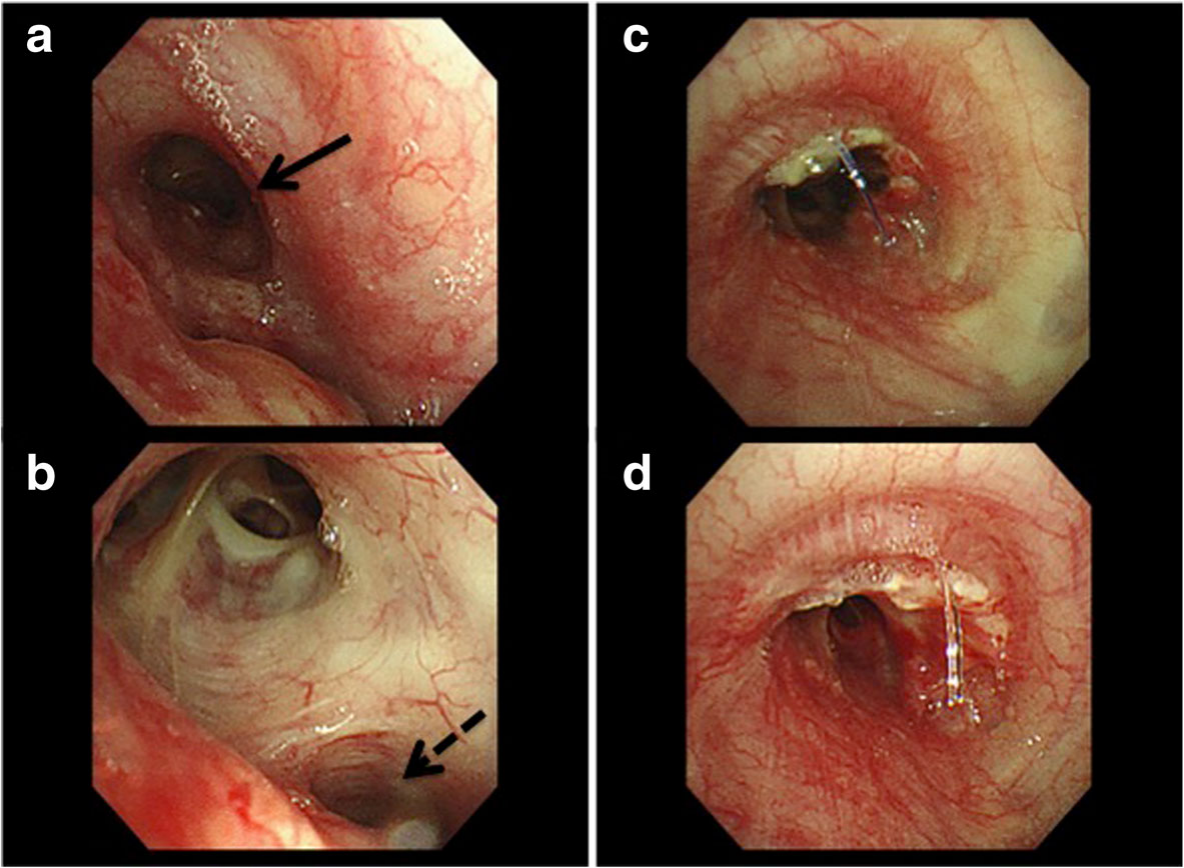
Preoperative bronchoscopy showed that tumor was exposed in the bronchial lumen and infiltrated to left main bronchus and upper bronchus (solid arrow) (**a**). Even though the scope could pass through the exposed tumor of upper bronchus, tumor also infiltrated to lingular division bronchus (dotted arrow) (**b**). Postoperative bronchoscopic findings showed no troubles at the anastomotic site including stenosis or kinking seven days after surgery (**c**) and one year after surgery (**d**)

**Fig. 3:**
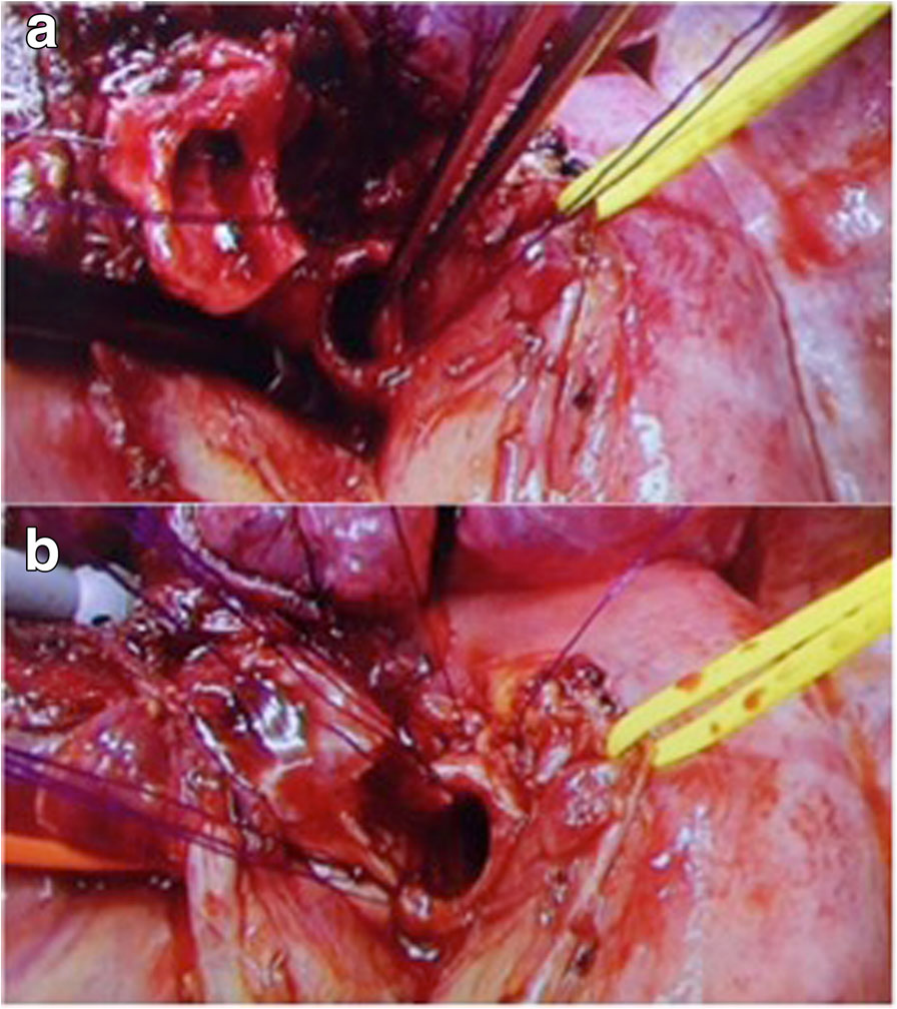
Reconstruction was performed with bronchial wall flap, “wine cup stoma” (**a** and **b**)

**Fig. 4:**
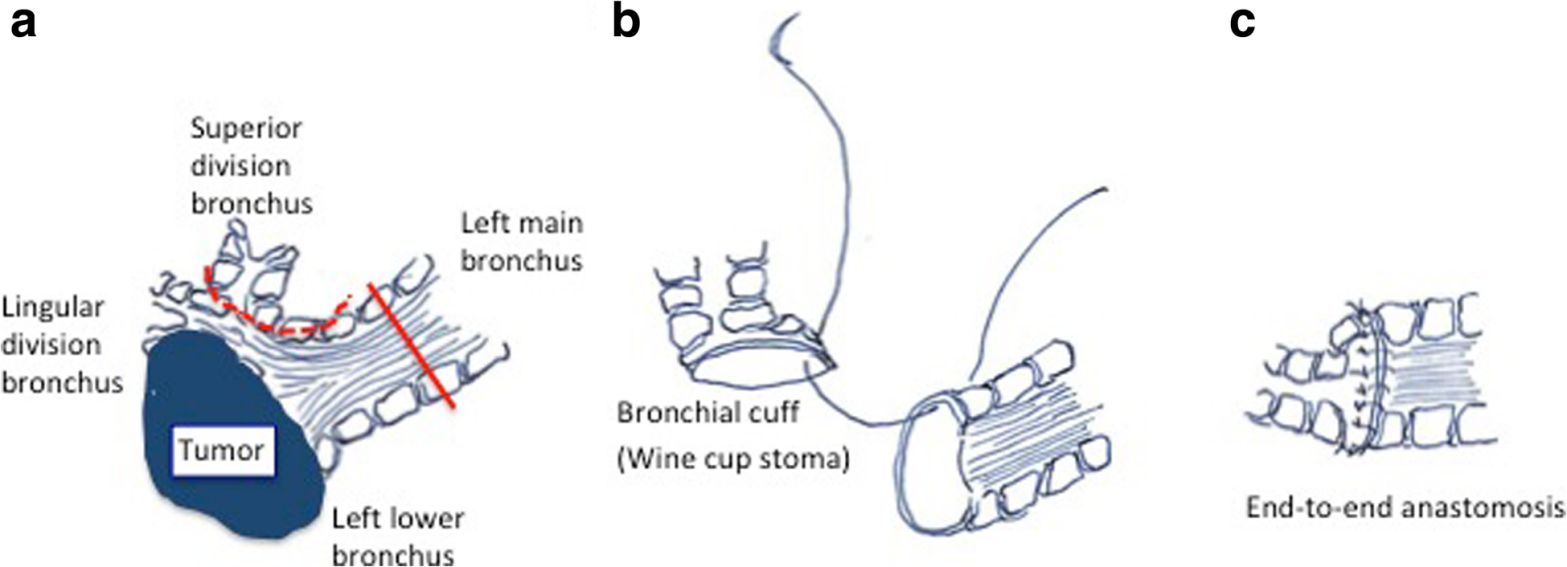
Scheme of procedure. Broken lines indicate the resection lines. The bronchus of the left superior division was edged with the partially excised wall of the left main bronchus to create cuff (dotted line) and left main bronchus was also transected (solid line) (**a**). End-to-end anastomosis was subsequently performed (**b** and **c**)

We report on a 64-year-old man suffering from hemoptysis, cough, mild fever and dyspnea. His computed tomography (CT) scan showed solid tumor of 40 mm in diameter in left lower bronchus (Fig. 1-a), which obstructed the lower bronchus and caused obstructive pneumonia of left lower lobe and expanded to second carina and pulmonary artery (Fig. 1-b). The CT scan also revealed severe pulmonary emphysema and his pulmonary function test showed obstructive function pattern (Table 1). His bronchoscopy showed that tumor was exposed in the bronchial lumen and infiltrated to left main bronchus and upper bronchus even though the scope could pass through the exposed tumor of upper bronchus (Fig. 2-a, b). Transbronchial lung biopsy showed squamous cell carcinoma. He had undergone left sleeve lingular segmentectomy and left lower lobectomy. The details of the procedure were as follows: a posterolateral thoracotomy at the fourth intercostal space was performed. The left lower lobe and lingular division were dissected. The resection point of bronchus was determined with almost 1 cm of the distance from tumor. Intraoperative pathological findings showed free surgical margin of the bronchus. Reconstruction was performed with bronchial wall flap using 4–0 PDS stitches (Johnson and Johnson K. K., NJ, US) (Fig. 3 and Fig. 4). The anastomotic site was wrapped using a fourth intercostal muscle flap. Although he had been suffered from prolonged air leakage due to alveolopleural fistula, he could discharge from our hospital one month after surgery. Pathological findings revealed moderately differentiated squamous cell carcinoma of pT3N0M0 stage IIB according to UICC 8th edition. Postoperative bronchoscopic findings showed no troubles at the anastomotic site including stenosis or kinking (Fig. 2-c, d). He had received no adjuvant chemotherapy after surgery because of his low pulmonary function. He has been well for eighteen months without any recurrences after surgery.

**Table 1:** Pulmonary function test (PFT) before surgery

VC	3020	ml	FEV1.0	1990	ml
%VC	87.0	%	FEV1.0%	63.5	%

VC: Vital capacity

FEV1.0: Forced expiratory volume in one second

## Discussion and conclusions

Lung cancer is the leading cause of cancer-related death worldwide [4]. Surgical resection is one of the mainstays for treatment of NSCLC together with chemotherapy, radiation therapy, and recent immunotherapy. Surgical treatment of NSCLC involving the proximal bronchi or PA can be challenging. Pneumonectomy is the most extensive pulmonary resection with which to ensure complete resection for these patients. However, pneumonectomy is associated with high complication rates, especially for patients with compromised pulmonary function. In recent years, the resectability of locally advanced lung cancer has been improving with advances in perioperative care, surgical techniques [5–7], and induction therapy [8–10], which downstages the tumors to render them resectable. Thus, avoidance of pneumonectomy can be achieved in selected patients at an early disease stage. The first sleeve lobectomy was performed by Prince-Thomas in 1942 [11], and the oncologic value of lobectomy with pulmonary arterioplasty was initially reported by Vogt-Moykopf et al. [12] in 1986. These procedures have since been accepted as valuable options to avoid pneumonectomy in selected patients. Many retrospective analyses have evaluated the operative mortality and morbidity of pneumonectomy and pulmonary function-preserving surgeries such as sleeve lobectomy [1–3] or PA reconstruction [13] in patients with NSCLC.

Previously, Okada and colleagues classified fifteen patients who underwent extended sleeve lobectomy into three groups according to the surgical procedure of reconstruction [14]. And Miyoshi and colleagues also reported three types of anastomotic techniques [15]. One is to use two adjusting stitches in the membranous part of the larger stump. The second technique is a telescoping anastomosis. The third technique is to make a cuff on the smaller stump by trimming the bronchus. Comparing with these procedures, the latter technique requires some adjustment of making cuff without remnant cancer cells. Before surgery, radiographic and endoscopic evaluations are needed to make a success of anastomosis. In this case, we planned to make a cuff using head-sided left main bronchus, which was cancer free side. Of course we should confirm and indeed had confirmed the pathological free margin during surgery. Okada and colleagues [14] described that resection points were determined with at least 1 cm of the macroscopically unaffected distance of the bronchus. We followed the resection point of this case according to this report [14]. Amazingly, this cuff technique was termed **“**wine cup stoma” by Maeda and colleagues [16] almost three decades ago and we called this simple procedure same as the above. This technique is relatively simple and postoperative complications such as anastomotic stenosis or kinking are avoidable. Toyooka and colleagues [17] also recommended this bronchial cuff technique rather than adjusting stitches.

For the indication of extended sleeve lobectomy, previous reports showed that invasion of the bronchus with N0 and N1 disease were the most suitable indication [18, 19]. According this recommendation, we performed type C extended sleeve lobectomy for this patient and achieved successful results to date.

In conclusions, we experienced a successful anastomosis of left sleeve lingular segmentectomy and lower lobectomy (type C extended sleeve lobectomy) with bronchial wall flap (wine cup stoma) for central-type lung cancer. This technique might be useful for other extended sleeve lobectomy and lung transplantation to avoid anastomotic complications.

## Abbreviations

CT: Computed Tomography
NSCLC: Non-small cell lung cancer
PA: Pulmonary artery
